# Supplementary material for: Septal secretion of protein A in Staphylococcus aureus requires SecA and lipoteichoic acid synthesis
Source: eLife. 2018 May 14;7:e34092. doi: 10.7554/eLife.34092 (PMC5962339; doi:10.7554/eLife.34092)
Supplement: Supplementary file 1. [file elife-34092-supp1.docx]

| **Table S1. List of ESI-MS identified tryptic peptides crosslinked to SpA_ED/S18L_** | | |
| --- | --- | --- |
| **Number of peptides** | **Gene Locus or Symbol** | **UniProt reference** |
| 26 | *secA* | (O06446_SECA_STAA8) |
| 21 | *alaS* | (Q2FXV9_SYA_STAA8) |
| 18 | *murG* | (Q2FYL5_MURG_STAA8) |
| 15 | *polA* | (Q2FXN9_Q2FXN9_STAA8) |
| 15 | SAOUHSC_01854 | (Q2G245_Q2G245_STAA8) |
| 15 | *femA* | (Q2FYR2_FEMA_STAA8) |
| 11 | *clpC* | (Q2G0P5_CLPC_STAA8) |
| 10 | SAOUHSC_01810 | (Q2FXM5_Q2FXM5_STAA8) |
| 10 | *ezrA* | (Q2FXK8_EZRA_STAA8) |
| 9 | *pbp2* | (Q2FYI0_Q2FYI0_STAA8) |
| 9 | *murE2* | (Q2FWZ9_Q2FWZ9_STAA8) |
| 9 | UDP-N-acetylglucosamine pyrophosphorylase | (Q2FW81_URTF_STAA8) |
| 9 | *femB* | (Q2FYR1_FEMB_STAA8) |
| 9 | *tagB* | (Q2G1C2_Q2G1C2_STAA8) |
| 8 | *tagF* | (Q2G1C1_Q2G1C1_STAA8) |
| 7 | *ltaS* | (Q2G093_LTAS_STAA8) |
| 7 | *femX* | (Q2FVZ4_FEMX_STAA8) |
| 6 | 1-acyl-sn-glycerol-3-phosphate acyltransferases domain protein | (Q2FXJ7_Q2FXJ7_STAA8) |
| 6 | *purL* | (Q2FZJ0_PURL_STAA8) |
| 6 | Conserved hypothetical phage protein | (Q2FY82_Q2FY82_STAA8) |
| 6 | SAOUHSC_02447 | (Q2G2D7_Q2G2D7_STAA8) |
| 6 | SAOUHSC_01347 | (Q2FYS9_Q2FYS9_STAA8) |
| 6 | SAOUHSC_01180 | (Q2G264_Q2G264_STAA8) |
| 5 | *clpB* | (Q2FZS8_Q2FZS8_STAA8) |
| 5 | *dltD* | (Q2FZW3_Q2FZW3_STAA8) |
| 5 | *cshA* | (Q2FWH5_Y2316_STAA8) |
| 5 | *prfC* | (Q2FZP4_RF3_STAA8) |
| 5 | *parE* | (Q2FYS5_PARE_STAA8) |
| 5 | *parC* | (Q2FYS4_PARC_STAA8) |
| 5 | *betA* | (Q2FV11_BETA_STAA8) |
| 5 | SAOUHSC_02417 | (Q2FW86_Q2FW86_STAA8) |
| 5 | SAOUHSC_02274 | (Q2FWL5_Q2FWL5_STAA8) |
| 5 | SAOUHSC_01960 | (Q2FXA5_Q2FXA5_STAA8) |
| 5 | SAOUHSC_01908 | (Q2G1W5_Q2G1W5_STAA8) |
| 5 | SAOUHSC_01613 | (Q2FY52_Q2FY52_STAA8) |
| 5 | SAOUHSC_01026 | (Q2FZH8_Q2FZH8_STAA8) |
| 5 | SAOUHSC_00309 | (Q2G146_Q2G146_STAA8) |
| 5 | SAOUHSC_00113 | (Q2G1K9_Q2G1K9_STAA8) |
| 4 | *saeS* | (Q2G2U1_SAES_STAA8) |
| 4 | *rplK* | (P0A0F4_RL11_STAA8) |
| 4 | *rplE* | (Q2FW18_RL5_STAA8) |
| 4 | *murC* | (Q2FXJ0_MURC_STAA8) |
| 4 | *metN2* | (Q2FZZ2_METN2_STAA8) |
| 4 | *lip2* | (Q2G155_LIP2_STAA8) |
| 4 | SAOUHSC_02859 | (Q2FV77_Q2FV77_STAA8) |
| 4 | SAOUHSC_02582 | (Q2FVV9_FDHL_STAA8) |
| 4 | SAOUHSC_02363 | (Q2FWD6_ALD1_STAA8) |
| 4 | SAOUHSC_01884 | (Q2FXG3_Q2FXG3_STAA8) |
| 4 | SAOUHSC_01855 | (Q2G247_Y1855_STAA8) |
| 4 | SAOUHSC_01723 | (Q2FXV8_Q2FXV8_STAA8) |
| 4 | SAOUHSC_01679 | (Q2FXZ6_Q2FXZ6_STAA8) |
| 4 | SAOUHSC_01460 | (Q2FYI6_Q2FYI6_STAA8) |
| 4 | SAOUHSC_01321 | (Q2FYV3_Q2FYV3_STAA8) |
| 4 | SAOUHSC_00893 | (Q2FZU7_Q2FZU7_STAA8) |
| 4 | SAOUHSC_00637 | (Q2G2L2_Q2G2L2_STAA8) |
| 4 | SAOUHSC_00531 | (Q2G0M9_Q2G0M9_STAA8) |
| 3 | *tgt* | (Q2FXT6_TGT_STAA8) |
| 3 | *queA* | (Q2FXT5_QUEA_STAA8) |
| 3 | *prs* | (Q2G0S2_Q2G0S2_STAA8) |
| 3 | *pcrA* | (Q53727_PCRA_STAA8) |
| 3 | *hemE* | (Q2FXA3_DCUP_STAA8) |
| 3 | *gyrA* | (Q2G2Q0_GYRA_STAA8) |
| 3 | *gpsA* | (Q2FYG1_GPDA_STAA8) |
| 3 | *glmU* | (Q2G0S3_GLMU_STAA8) |
| 3 | SAOUHSC_02684 | (Q2FVL8_Q2FVL8_STAA8) |
| 3 | SAOUHSC_02627 | (Q2G2W3_Q2G2W3_STAA8) |
| 3 | SAOUHSC_02317 | (Q2FWH4_Q2FWH4_STAA8) |
| 3 | SAOUHSC_02134 | (Q2G234_Q2G234_STAA8) |
| 3 | SAOUHSC_01973 | (Q2G2T1_Q2G2T1_STAA8) |
| 3 | SAOUHSC_01728 | (Q2FXV3_Q2FXV3_STAA8) |
| 3 | SAOUHSC_01673 | (Q2FY01_Q2FY01_STAA8) |
| 3 | SAOUHSC_01612 | (Q2FY53_Q2FY53_STAA8) |
| 3 | SAOUHSC_01584 | (Q2FY81_Q2FY81_STAA8) |
| 3 | SAOUHSC_01499 | (Q2FYF3_Q2FYF3_STAA8) |
| 3 | SAOUHSC_01490 | (Q2FYG2_Q2FYG2_STAA8) |
| 3 | SAOUHSC_01071 | (Q2FZF9_Q2FZF9_STAA8) |
| 3 | SAOUHSC_00875 | (Q2FZW0_Q2FZW0_STAA8) |
| 3 | SAOUHSC_00756 | (Q2G065_Q2G065_STAA8) |
| 3 | SAOUHSC_00731 | (Q2G089_Q2G089_STAA8) |
| 3 | SAOUHSC_00584 | (Q2G0I1_Q2G0I1_STAA8) |
| 3 | SAOUHSC_00480 | (Q2G0R6_Q2G0R6_STAA8) |
| 3 | SAOUHSC_00467 | (Q2G0S7_Q2G0S7_STAA8) |
| 3 | SAOUHSC_00139 | (Q2G1I3_Q2G1I3_STAA8) |
| 2 | *pknB* | (Q2FZ64_Q2FZ64_STAA8) |
| 2 | *tsaD* | (Q2FWL2_TSAD_STAA8) |
| 2 | *topB* | (Q2FW03_TOP3_STAA8) |
| 2 | *topA* | (Q2FZ32_TOP1_STAA8) |
| 2 | *rsmH* | (P60393_RSMH_STAA8) |
| 2 | *rsmA* | (Q2G0T0_RSMA_STAA8) |
| 2 | *rpsG* | (P48940_RS7_STAA8) |
| 2 | *rpsC* | (Q2FW12_RS3_STAA8) |
| 2 | *rplM* | (Q2FW38_RL13_STAA8) |
| 2 | *rplI* | (Q2G2T3_RL9_STAA8) |
| 2 | *rplF* | (Q2FW21_RL6_STAA8) |
| 2 | *potA* | (Q2G2A7_POTA_STAA8) |
| 2 | *mutS* | (Q2FYZ9_MUTS_STAA8) |
| 2 | *murE* | (Q2FZP6_MURE_STAA8) |
| 2 | *lysA* | (Q2FYN4_Q2FYN4_STAA8) |
| 2 | *gcvT* | (Q2FY33_GCST_STAA8) |
| 2 | *fmt* | (Q2FZ68_FMT_STAA8) |
| 2 | *ebpS* | (Q2FYF1_EBPS_STAA8) |
| 2 | *dnaJ* | (Q2FXZ3_DNAJ_STAA8) |
| 2 | *atpG* | (Q2FWE9_Q2FWE9_STAA8) |
| 2 | *aroC* | (Q2FYG9_AROC_STAA8) |
| 2 | *alr1* | (Q9ZAH5_ALR1_STAA8) |
| 2 | *ald2* | (Q2FXL7_DHA2_STAA8) |
| 2 | SAOUHSC_02980 | (Q2G220_Q2G220_STAA8) |
| 2 | SAOUHSC_02899 | (Q2FV40_Q2FV40_STAA8) |
| 2 | SAOUHSC_02875 | (Q2FV62_Q2FV62_STAA8) |
| 2 | SAOUHSC_02382 | (Q2FWB6_Q2FWB6_STAA8) |
| 2 | SAOUHSC_02145 | (Q2FWX6_Q2FWX6_STAA8) |
| 2 | SAOUHSC_02133 | (Q2G235_Q2G235_STAA8) |
| 2 | SAOUHSC_01998 | (Q2G281_Q2G281_STAA8) |
| 2 | SAOUHSC_01816 | (Q2FXL9_Y1816_STAA8) |
| 2 | SAOUHSC_01794 | (Q2FXP2_Q2FXP2_STAA8) |
| 2 | SAOUHSC_01791 | (Q2FXP5_Q2FXP5_STAA8) |
| 2 | SAOUHSC_01766 | (Q2FXR9_Q2FXR9_STAA8) |
| 2 | SAOUHSC_01660 | (Q2FY14_Q2FY14_STAA8) |
| 2 | SAOUHSC_01615 | (Q2FY50_Q2FY50_STAA8) |
| 2 | SAOUHSC_01606 | (Q2FY59_Q2FY59_STAA8) |
| 2 | SAOUHSC_01486 | (Q2FYG6_Q2FYG6_STAA8) |
| 2 | SAOUHSC_01249 | (Q2G2Q2_Q2G2Q2_STAA8) |
| 2 | SAOUHSC_01199 | (Q2FZ53_Q2FZ53_STAA8) |
| 2 | SAOUHSC_01184 | (Q2FZ67_Q2FZ67_STAA8) |
| 2 | SAOUHSC_01054 | (Q2G2G7_Y1054_STAA8) |
| 2 | SAOUHSC_01014 | (Q2FZI9_Q2FZI9_STAA8) |
| 2 | SAOUHSC_00834 | (Q2G000_Q2G000_STAA8) |
| 2 | SAOUHSC_00794 | (Q2G033_Q2G033_STAA8) |
| 2 | SAOUHSC_00508 | (Q2G242_Q2G242_STAA8) |
| 2 | SAOUHSC_00442 | (Q2G0T5_Q2G0T5_STAA8) |
| 1 | yajC | (Q2FXT7_Q2FXT7_STAA8) |
| 1 | *secDF* | (Q2FXT8_Q2FXT8_STAA8) |
| 1 | *scaH* | (Q2G222_Y2979_STAA8) |
| 1 | *murI* | (Q2FZC6_MURI_STAA8) |
| 1 | *msrR* | (Q7BHL7_MSRR_STAA8) |
| 1 | *msrB* | (P0A088_MSRB_STAA8) |
| 1 | *xerD* | (Q2FY74_Q2FY74_STAA8) |
| 1 | *uvrC* | (Q2FZD0_UVRC_STAA8) |
| 1 | *uvrA* | (Q2G046_Q2G046_STAA8) |
| 1 | *ugtP* | (Q2FZP7_UGTP_STAA8) |
| 1 | *tagX* | (O05154_TAGX_STAA8) |
| 1 | *srrB* | (Q2FY80_SRRB_STAA8) |
| 1 | *sarZ* | (Q2FVN3_SARZ_STAA8) |
| 1 | *sarS* | (Q2G1N7_SARS_STAA8) |
| 1 | *sarR* | (Q9F0R1_SARR_STAA8) |
| 1 | *sarA* | (Q2G2U9_SARA_STAA8) |
| 1 | *gdpp* | (Q2G2T6_Q2G2T6_STAA8) |
| 1 | *ruvB* | (Q2FXT4_RUVB_STAA8) |
| 1 | *rpsL* | (P0A0H0_RS12_STAA8) |
| 1 | *rpsD* | (Q2FXK6_RS4_STAA8) |
| 1 | *rpmI* | (Q2FXQ0_RL35_STAA8) |
| 1 | *rplW* | (Q2FW08_RL23_STAA8) |
| 1 | *rplP* | (Q2FW13_RL16_STAA8) |
| 1 | *rplN* | (Q2FW16_RL14_STAA8) |
| 1 | *rot* | (Q9RFJ6_ROT_STAA8) |
| 1 | *rnr* | (Q2G024_Q2G024_STAA8) |
| 1 | *recG* | (O50581_RECG_STAA8) |
| 1 | *pyrF* | (Q2FZ71_PYRF_STAA8) |
| 1 | *putP* | (Q2FWY7_PUTP_STAA8) |
| 1 | *nusG* | (Q2G0P2_NUSG_STAA8) |
| 1 | *mutS2* | (Q2FZD3_MUTS2_STAA8) |
| 1 | *mqo* | (Q2FVQ5_Q2FVQ5_STAA8) |
| 1 | *moaA* | (P69848_MOAA_STAA8) |
| 1 | *infC* | (Q2FXP9_IF3_STAA8) |
| 1 | *hslO* | (Q2G0Q9_HSLO_STAA8) |
| 1 | *hemH* | (Q2FXA4_Q2FXA4_STAA8) |
| 1 | *guaC* | (Q2FYU4_GUAC_STAA8) |
| 1 | *gmk* | (Q2G1U0_KGUA_STAA8) |
| 1 | *ftsY* | (Q2FZ48_Q2FZ48_STAA8) |
| 1 | *dltC* | (Q2FZW4_DLTC_STAA8) |
| 1 | *dinG* | (Q2FYH5_DING_STAA8) |
| 1 | *cvfB* | (Q2FYP3_CVFB_STAA8) |
| 1 | *copA* | (Q2FV64_COPA_STAA8) |
| 1 | *cinA* | (Q2FZ10_Q2FZ10_STAA8) |
| 1 | *bioA* | (Q2FVJ6_BIOA_STAA8) |
| 1 | *atpF* | (Q2G2F8_ATPF_STAA8) |
| 1 | *addA* | (Q2FZT5_ADDA_STAA8) |
| 1 | SAOUHSC_02525 (RND2) | (Q2FVZ5_Q2FVZ5_STAA8) |
| 1 | SAOUHSC_03016 | (Q2FUT5_Q2FUT5_STAA8) |
| 1 | SAOUHSC_02971 | (Q2FUX4_Q2FUX4_STAA8) |
| 1 | SAOUHSC_02956 | (Q2FUY9_Q2FUY9_STAA8) |
| 1 | SAOUHSC_02947 | (Q2FUZ8_Q2FUZ8_STAA8) |
| 1 | SAOUHSC_02791 | (Q2FVC2_Q2FVC2_STAA8) |
| 1 | SAOUHSC_02760 | (Q2FVF4_Q2FVF4_STAA8) |
| 1 | SAOUHSC_02727 | (Q2FVI3_Q2FVI3_STAA8) |
| 1 | SAOUHSC_02723 | (Q2FVI7_Q2FVI7_STAA8) |
| 1 | SAOUHSC_02690 | (Q2G1U8_Q2G1U8_STAA8) |
| 1 | SAOUHSC_02681 | (Q2FVM1_Q2FVM1_STAA8) |
| 1 | SAOUHSC_02668 | (Q2FVN4_Q2FVN4_STAA8) |
| 1 | SAOUHSC_02660 | (Q2FVP2_Q2FVP2_STAA8) |
| 1 | SAOUHSC_02649 | (Q2FVQ3_Q2FVQ3_STAA8) |
| 1 | SAOUHSC_02648 | (Q2FVQ4_Q2FVQ4_STAA8) |
| 1 | SAOUHSC_02629 | (Q2G2W1_Q2G2W1_STAA8) |
| 1 | SAOUHSC_02614 | (Q2FVS7_Q2FVS7_STAA8) |
| 1 | SAOUHSC_02601 | (Q2FVU1_Q2FVU1_STAA8) |
| 1 | SAOUHSC_02583 | (Q2FVV8_Q2FVV8_STAA8) |
| 1 | SAOUHSC_02555 | (Q2FVW8_Q2FVW8_STAA8) |
| 1 | SAOUHSC_02554 | (Q2FVW9_Q2FVW9_STAA8) |
| 1 | SAOUHSC_02553 | (Q2FVX0_Q2FVX0_STAA8) |
| 1 | SAOUHSC_02544 | (Q2FVX8_Q2FVX8_STAA8) |
| 1 | SAOUHSC_02406 | (Q2FW93_Q2FW93_STAA8) |
| 1 | SAOUHSC_02381 | (Q2FWB7_Q2FWB7_STAA8) |
| 1 | SAOUHSC_02374 | (Q2FWC4_Q2FWC4_STAA8) |
| 1 | SAOUHSC_02357 | (Q2FWE2_Q2FWE2_STAA8) |
| 1 | SAOUHSC_02352 | (Q2G2F5_Q2G2F5_STAA8) |
| 1 | SAOUHSC_02197 | (Q2FWT3_Q2FWT3_STAA8) |
| 1 | SAOUHSC_02161 | (Q2FWW1_Q2FWW1_STAA8) |
| 1 | SAOUHSC_02098 | (Q2FX09_Q2FX09_STAA8) |
| 1 | SAOUHSC_01987 | (Q2FX90_Q2FX90_STAA8) |
| 1 | SAOUHSC_01979 | (Q2FX98_Q2FX98_STAA8) |
| 1 | SAOUHSC_01978 | (Q2FX99_Y1978_STAA8) |
| 1 | SAOUHSC_01977 | (Q2FXA0_Y1977_STAA8) |
| 1 | SAOUHSC_01969 | (Q2G2T0_Q2G2T0_STAA8) |
| 1 | SAOUHSC_01966 | (Q2G2F2_Q2G2F2_STAA8) |
| 1 | SAOUHSC_01915 | (Q2G2V2_Q2G2V2_STAA8) |
| 1 | SAOUHSC_01877 | (Q2FXH0_Q2FXH0_STAA8) |
| 1 | SAOUHSC_01869 | (Q2FXH8_Q2FXH8_STAA8) |
| 1 | SAOUHSC_01867 | (Q2FXI0_Q2FXI0_STAA8) |
| 1 | SAOUHSC_01846 | (Q2G294_Q2G294_STAA8) |
| 1 | SAOUHSC_01825 | (Q2FXL0_Q2FXL0_STAA8) |
| 1 | SAOUHSC_01812 | (Q2FXM3_Q2FXM3_STAA8) |
| 1 | SAOUHSC_01803 | (Q2FXN2_Q2FXN2_STAA8) |
| 1 | SAOUHSC_01801 | (Q2FXN4_Q2FXN4_STAA8) |
| 1 | SAOUHSC_01744 | (Q2FXT9_Q2FXT9_STAA8) |
| 1 | SAOUHSC_01734 | (Q2FXU8_Q2FXU8_STAA8) |
| 1 | SAOUHSC_01732 | (Q2FXV0_Q2FXV0_STAA8) |
| 1 | SAOUHSC_01700 | (Q2FXY0_Q2FXY0_STAA8) |
| 1 | SAOUHSC_01664 | (Q2FY10_PDRP_STAA8) |
| 1 | SAOUHSC_01659 | (Q2FY15_Q2FY15_STAA8) |
| 1 | SAOUHSC_01652 | (Q2FY21_Q2FY21_STAA8) |
| 1 | SAOUHSC_01610 | (Q2FY55_Y1610_STAA8) |
| 1 | SAOUHSC_01587 | (Q2FY78_Q2FY78_STAA8) |
| 1 | SAOUHSC_01488 | (Q2FYG4_Q2FYG4_STAA8) |
| 1 | SAOUHSC_01487 | (Q2FYG5_Q2FYG5_STAA8) |
| 1 | SAOUHSC_01480 | (Q2FYH2_Q2FYH2_STAA8) |
| 1 | SAOUHSC_01455 | (Q2FYJ0_Q2FYJ0_STAA8) |
| 1 | SAOUHSC_01436 | (Q2FYK4_Y1436_STAA8) |
| 1 | SAOUHSC_01284 | (Q2FYY8_Q2FYY8_STAA8) |
| 1 | SAOUHSC_01279 | (Q2FYZ3_Q2FYZ3_STAA8) |
| 1 | SAOUHSC_01267 | (Q2FZ04_Q2FZ04_STAA8) |
| 1 | SAOUHSC_01258 | (Q2FZ13_Q2FZ13_STAA8) |
| 1 | SAOUHSC_01214 | (Q2FZ39_Q2FZ39_STAA8) |
| 1 | SAOUHSC_01198 | (Q2FZ54_Q2FZ54_STAA8) |
| 1 | SAOUHSC_01179 | (Q2G266_Q2G266_STAA8) |
| 1 | SAOUHSC_01031 | (Q2FZH3_Q2FZH3_STAA8) |
| 1 | SAOUHSC_01016 | (Q2FZI7_Q2FZI7_STAA8) |
| 1 | SAOUHSC_00989 | (Q2FZL1_Q2FZL1_STAA8) |
| 1 | SAOUHSC_00982 | (Q2FZL8_Q2FZL8_STAA8) |
| 1 | SAOUHSC_00974 | (Q2FZM6_Q2FZM6_STAA8) |
| 1 | SAOUHSC_00951 | (Q2FZP9_Y951_STAA8) |
| 1 | SAOUHSC_00946 | (Q2FZQ4_Q2FZQ4_STAA8) |
| 1 | SAOUHSC_00925 | (Q2FZR5_Q2FZR5_STAA8) |
| 1 | SAOUHSC_00909 | (Q2FZT1_Q2FZT1_STAA8) |
| 1 | SAOUHSC_00897 | (Q2FZU3_Q2FZU3_STAA8) |
| 1 | SAOUHSC_00873 | (Q2FZW2_Q2FZW2_STAA8) |
| 1 | SAOUHSC_00855 | (Q2FZX9_2NPD_STAA8) |
| 1 | SAOUHSC_00847 | (Q2FZY7_Q2FZY7_STAA8) |
| 1 | SAOUHSC_00792 | (Q2G035_Y792_STAA8) |
| 1 | SAOUHSC_00730 | (Q2G090_Q2G090_STAA8) |
| 1 | SAOUHSC_00727 | (Q2G094_Q2G094_STAA8) |
| 1 | SAOUHSC_00711 | (Q2G2T9_Q2G2T9_STAA8) |
| 1 | SAOUHSC_00707 | (Q2G238_Q2G238_STAA8) |
| 1 | SAOUHSC_00640 | (Q2G2L3_Q2G2L3_STAA8) |
| 1 | SAOUHSC_00639 | (Q2G2K9_Q2G2K9_STAA8) |
| 1 | SAOUHSC_00547 | (Q2G0L3_Q2G0L3_STAA8) |
| 1 | SAOUHSC_00483 | (Q2G0R3_Q2G0R3_STAA8) |
| 1 | SAOUHSC_00444 | (Q2G0T4_Y444_STAA8) |
| 1 | SAOUHSC_00413 | (Q2G0W1_Y413_STAA8) |
| 1 | SAOUHSC_00398 | (Q2G0X5_Q2G0X5_STAA8) |
| 1 | SAOUHSC_00333 | (Q2G1V4_Q2G1V4_STAA8) |
| 1 | SAOUHSC_00307 | (Q2G148_Q2G148_STAA8) |
| 1 | SAOUHSC_00269 | (Q2G178_Q2G178_STAA8) |
| 1 | SAOUHSC_00268 | (Q2G179_Q2G179_STAA8) |
| 1 | SAOUHSC_00261 | (Q2G185_Q2G185_STAA8) |
| 1 | SAOUHSC_00236 | (Q2G1A9_Q2G1A9_STAA8) |
| 1 | SAOUHSC_00196 | (Q2G1C9_Q2G1C9_STAA8) |
| 1 | SAOUHSC_00126 | (Q2G1J6_Q2G1J6_STAA8) |
| 1 | SAOUHSC_00039 | (Q2G1R3_Q2G1R3_STAA8) |
