# Supplementary material for: Septal secretion of protein A in Staphylococcus aureus requires SecA and lipoteichoic acid synthesis
Source: eLife. 2018 May 14;7:e34092. doi: 10.7554/eLife.34092 (PMC5962339; doi:10.7554/eLife.34092)
Supplement: Supplementary file 2. [file elife-34092-supp2.docx]

| **Table S2. Strains and plasmids used in this study** | | |
| --- | --- | --- |
| **Strain or plasmid** | **Description** | **Reference or source** |
| *E. coli* DC10B | Cloning strain | (Monk et al., 2012) |
| *E. coli* DH5α | Cloning strain | (Hanahan, 1983) |
| *S. aureus* RN4220 | *S. aureus* laboratory strain | (Kreiswirth et al., 1983) |
| *S. aureus* SEJ1 | ∆*spa* in RN4220 | (Gründling and Schneewind, 2007) |
| *S. aureus* WY110 | ∆*spa* ∆*sbi, sbi*::*ermB* in *S. aureus* SEJ1 | This work |
| *S. aureus* WY223 | P*_spac_-secA* in *S. aureus* RN4220 | This work |
| *S. aureus* WY230 | P*_tet_-secA:sfGFP* in *S. aureus* WY223 | This work |
| *S. aureus* ANG499 | P*_spac_-ltaS* in *S. aureus* RN4220 | (Gründling and Schneewind, 2007) |
| *S. aureus* WY418 | ∆*secDF* in *S. aureus* RN4220 | This work |
| *S. aureus* WY416 | ∆*rnd2* in *S. aureus* RN4220 | This work |
| *S. aureus* WY400 | ∆*rnd3* in *S. aureus* RN4220 | This work |
| *S. aureus* WY412 | ∆*secDF* ∆*rnd2*∆*rnd3* in *S. aureus* RN4220 | This work |
| pOS1 | *E. coli/S. aureus* shuttle vector | (Schneewind et al., 1993) |
| pSpA_ED_ | *spa* promoter, signal peptide and IgBDs E and D in pOS1 | This work |
| pSpA_ED/R10A_ | R10A variant of pSpA_ED_ | This work |
| pSpA_ED/S18L_ | S18L variant of of pSpA_ED_ | This work |
| pSpA_ED/ΔIA_ | ∆IA variant of pSpA_ED_ | This work |
| pCL55 | *S. aureus* integration vector | (Lee et al., 1991) |
| pCL55-SpA | Full length *spa* with its native promoter cloned in pCL55 | This work |
| pCL55-SpA_R10A_ | R10A variant of pCL55-SpA | This work |
| pCL55-SpA_S18L_ | S18L variant of pCL55-SpA | This work |
| pCL55-SpA_ΔIA_ | ∆IA variant of pCL55-SpA | This work |
| pCL55-SpA_SP-SasF_ | SpA signal peptide replaced by SasF signal peptide in pCL55-SpA | This work |
| pCL55-P*_tet_* | pCL55 with anhydrotetracycline inducible promoter | (Gründling and Schneewind, 2007) |
| pCL55-P*_tet_*-*secA:sfGFP* | SecA-sfGFP hybrid cloned into pCL55-P*_tet_* | This work |
| pMutin–HA | Single copy integration vector | Bacillus Genetic Stock Center |
| pMutin–HA-5’secA | *secA* promoter and 656 bp in pMutin-HA | This work |
| pKOR1-*secDF* | allelic replacement vector for *secDF* deletion | This work |
| pKOR1-*rnd2* | allelic replacement vector for *rnd2* deletion | This work |
| pSecDF | *secDF* ORF and 274 bp upstream in pOS1 | This work |

**REFERENCES**

HANAHAN, D. 1983. Studies on transformation of *Escherichia coli* with plasmids. *J Mol Biol,* 166**,** 557-80.

KREISWIRTH, B. N., LOFDAHL, S., BETLEY, M. J., O'REILLY, M., SCHLIEVERT, P. M., BERGDOLL, M. S. & NOVICK, R. P. 1983. The toxic shock syndrome exotoxin structural gene is not detectably transmitted by a prophage. *Nature,* 305**,** 709-12.
