# Supplementary material for: Septal secretion of protein A in Staphylococcus aureus requires SecA and lipoteichoic acid synthesis
Source: eLife. 2018 May 14;7:e34092. doi: 10.7554/eLife.34092 (PMC5962339; doi:10.7554/eLife.34092)
Supplement: Supplementary file 3. [file elife-34092-supp3.docx]

| **Table S3. Oligonucleotide primers used in this study** | | | | |
| --- | --- | --- | --- | --- |
| **Primer** | **^a^Direction** | | **Sequence** | **Plasmid** |
| 10 | F | GCGTAGTATTGCAATACATAATTCGTTA | | pSpA_ED_ |
| 69 | R | TTTTGGATCCTTACATTTTCGGTGCTTGAGATTCGTT | | pSpA_ED_ |
| 38 | R | TGCAATACCTACACCTAGAATGTTTTTCTTTTTCA | | pSpA_ED/∆YSIRK_ |
| 39 | F | GAAAAAGAAAAACATTCTAGGTGTAGGTATTGCATC | | pSpA_ED/∆YSIRK_ |
| 49 | R | TGTACCTAAAGTTACTACACCTAGTTTACGA | | pSpA_ED/∆GIAS_ |
| 50 | F | TCGTAAACTAGGTGTAGTAACTTTAGGTACA | | pSpA_ED/∆GIAS_ |
| 51 | R | TGTACCTAAAGTTACTGCAATTACACCTAGT | | pSpA_ED/∆G15∆S18_ |
| 52 | F | ACTAGGTGTAATTGCAGTAACTTTAGGTACA | | pSpA_ED/∆G15∆S18_ |
| 53 | R | ACCTAAAGTTACAGAACCTACACCTAGTTTAC | | pSpA_ED/∆IA_ |
| 54 | F | GTAAACTAGGTGTAGGTTCTGTAACTTTAGGT | | pSpA_ED/∆IA_ |
| 55 | R | AGTTACAGATGCAATTAATACACCTAGTTTACGA | | pSpA_ED/G15L_ |
| 56 | F | TCGTAAACTAGGTGTATTAATTGCATCTGTAACT | | pSpA_ED/G15L_ |
| 57 | R | TGTACCTAAAGTTACTAATGCAATACCTACACCT | | pSpA_ED/S18L_ |
| 58 | F | AGGTGTAGGTATTGCATTAGTAACTTTAGGTACA | | pSpA_ED/S18L_ |
| 70 | R | AGTAATGTACCTAAAGTTACCAATGCAATTAATACACCTAGT | | pSpA_ED/G15L/S18L_ |
| 71 | F | ACTAGGTGTATTAATTGCATTGGTAACTTTAGGTACATTACT | | pSpA_ED/G15L/S18L_ |
| 72 | R | AGTAATGTACCTAAAGTTACAGATGCAATTACACCTAGTTTACGA | | pSpA_ED/∆G15_ |
| 73 | F | TCGTAAACTAGGTGTAATTGCATCTGTAACTTTAGGTACATTACT | | pSpA_ED/∆G15_ |
| 74 | R | ACCTAAAGTTACTGCAATACCTACACCTAGTTTACGAATTGA | | pSpA_ED/∆S18_ |
| 75 | F | TCAATTCGTAAACTAGGTGTAGGTATTGCAGTAACTTTAGGT | | pSpA_ED/∆S18_ |
| 84 | R | ATACCTACACCTAGTTTAGCAATTGAATAAATGTTT | | pSpA_ED/R10A_ |
| 85 | F | AAACATTTATTCAATTGCTAAACTAGGTGTAGGTAT | | pSpA_ED/R10A_ |
| 92 | R | TAGTTTACGAATTGAAGCAATGTTTTTCTTTTTCA | | pSpA_ED/Y7A_ |
| 93 | F | TGAAAAAGAAAAACATTGCTTCAATTCGTAAACTA | | pSpA_ED/Y7A_ |
| 94 | R | TACACCTAGTTTACGAGATGAATAAATGTTTTTCT | | pSpA_ED/I9S_ |
| 95 | F | AGAAAAACATTTATTCATCTCGTAAACTAGGTGTA | | pSpA_ED/I9S_ |
| 96 | R | TGCAATACCTACACCTAGAGCACGAATTGAATAAATGT | | pSpA_ED/K11A_ |
| 97 | F | ACATTTATTCAATTCGTGCTCTAGGTGTAGGTATTGCA | | pSpA_ED/K11A_ |
| 175 | F | GCGGGATCCTAGTATTGCAATACATAATTCGTTA | | pCL55-SpA |
| 177 | R | GCGGGTACCTTATAGTTCGCGACGACGTCCAGCT | | pCL55-SpA |
| 21 | R | ATTAATACCCCCTGTATGTATTTGT | | pCL55-SpA_SP-SasF_ |
| 22 | F | TACAGGGGGTATTAATATGGCTAAATATCGAGGGAAAC | | pCL55-SpA_SP-SasF_ |
| 23 | R | TGTTGAGCTTCATCGTGTTGCGCAGCTTGGGCATCGTACGGCAAGA | | pCL55-SpA_SP-SasF_ |
| 24 | F | GCGCAACACGATGAAGCTCAACAA | | pCL55-SpA_SP-SasF_ |
| 189 | F | CCCAAGCTTTAGCTAAAGGAGCGAACGAAATGGGA | | pMUTIN-HA-5’*secA* |
| 190 | R | GCGGGTACCTGAGTCAACCTCATCAATGATTGCA | | pMUTIN-HA-5’*secA* |
| 180 | F | CTCCCTAGGTAAAGGAGCGAACGAAATGGGAT | | pCL55-P_tet-_*secA:sfGFP* |
| 181 | R | TGCAGCTCCTGCGGCGCCTCCTTTTCCATGGCAATTTTTGA | | pCL55-P_tet-_*secA:sfGFP* |
| 182 | F | AGGAGGCGCCGCAGGAGCTGCATCAAAAGGTGAAGAATT | | pCL55-P_tet-_*secA:sfGFP* |
| 183 | R | CTCAGATCTTTATTTATATAATTCATCCATACCA | | pCL55-P_tet-_*secA:sfGFP* |
| 295 | F | GGGGACAAGTTTGTACAAAAAAGCAGGCTAATATTGTCATTGTATCCCGCTTCT | | pKOR1-*secDF* |
| 313 | R | ACATACGTAAATATCGAACGATGAAAAGATTTTAGT | | pKOR1-*secDF* |
| 314 | F | TCATCGTTCGATATTTACGTATGTATTTAGAATACT | | pKOR1-*secDF* |
| 298 | R | GGGGACCACTTTGTACAAGAAAGCTGGGTTGAACATACAGAGCAGTTTATGCCT | | pKOR1-*secDF* |
| 305 | F | GGGGACAAGTTTGTACAAAAAAGCAGGCTACATACTCCACAGATATTTTAGA | | pKOR1-*rnd2* |
| 306 | R | TGAATATAGATAATATAAAAGCCATAAAAGCGGT | | pKOR1-*rnd2* |
| 307 | F | TGGCTTTTATATTATCTATATTCAAAAATATTTTACT | | pKOR1-*rnd2* |
| 308 | R | GGGGACCACTTTGTACAAGAAAGCTGGGTTCGATCCTGATGTTGAAGTTGAT | | pKOR1-*rnd2* |
| 315 | F | GCGGAATTCTGAGAAGTGGTATTAAAAAGGATGA | | pSecDF |
| 316 | R | GCGGGATCCTTAAACTAAAATCTTTTCATCGTTCGA | | pSecDF |
| ^a^PCR primer direction for forward (F) or reverse (R) amplification of template DNA. | | | | |
